# *Clostridium difficile* Infections among Hospitalized Children, United States, 1997–2006

**DOI:** 10.3201/eid1604.090680

**Published:** 2010-04

**Authors:** Marya D. Zilberberg, Glenn S. Tillotson, L. Clifford McDonald

**Affiliations:** University of Massachusetts, Amherst, Massachusetts, USA (M.D. Zilberberg); Evi*Med* Research Group, LLC, Goshen, Massachusetts, USA (M.D. Zilberberg); ViroPharma Inc., Exton, Pennsylvania, USA (G.S. Tillotson); Centers for Disease Control and Prevention, Atlanta, Georgia, USA (L.C. McDonald)

**Keywords:** Clostridium difficile, bacteria, pediatric, hospital, children, epidemiology, United States, research

## Abstract

We evaluated the annual rate (cases/10,000 hospitalizations) of pediatric hospitalizations with *Clostridium difficile* infection (CDI; International Classification of Diseases, 9th revision, clinical modification code 008.45) in the United States. We performed a time-series analysis of data from the Kids’ Inpatient Database within the Health Care Cost and Utilization Project during 1997–2006 and a cross-sectional analysis within the National Hospital Discharge Survey during 2006. The rate of pediatric CDI-related hospitalizations increased from 7.24 to 12.80 from 1997 through 2006; the lowest rate was for children <1 year of age. Although incidence was lowest for newborns (0.5), incidence for children <1 year of age who were not newborns (32.01) was similar to that for children 5–9 years of age (35.27), which in turn was second only to incidence for children 1–4 years of age (44.87). Pediatric CDI-related hospitalizations are increasing. A better understanding of the epidemiology and outcomes of CDI is urgently needed.

## Abstract 2

Physicians need a better understanding of outcomes of these infections.

The epidemiology of *Clostridium difficile* infection (CDI) has been shifting over the past decade. Since 2000, the molecular evolution of the hypervirulent toxigenic bacterial strain BI/NAP1/027, which causes severe disease in massive outbreak settings, has been well documented (*1*–*4*). Furthermore, the increasing detection of this strain in the United States and other countries coincides with reports of increasing hospitalizations either resulting from or complicated by CDI and associated with increased case-fatality rates (*5*–*7*). Although in the past it was not thought to affect pediatric populations substantially, CDI has more recently been implicated as an increasingly prevalent diarrheal pathogen in children (*8*–*10*). Moreover, evidence suggests that a large proportion of pediatric CDI cases are community-acquired infections and that many of these infections lack the traditional risk factor of exposure to antimicrobial drugs (*11*–*13*). These changes in the epidemiology of pediatric CDI, although not definitively caused by the BI/NAP1/027 strain, are likely related to this strain because at least 2 reports suggest a high prevalence (10%–38%) of this strain in pediatric CDI populations and a 4× increase in complication rates associated with this strain compared with other strains (*14*,*15*).

Current age-specific epidemiology of CDI among children remains poorly studied. Literature predating the emergence of the epidemic strain suggests that although up to 67% of all neonates (i.e., <1 month of age) become colonized with *C*. *difficile* in the perinatal period, they do not appear to be at risk for the development of CDI-associated symptoms (*16*). Conversely, children 1 month–2 years of age, although less likely to become colonized with this bacterium, are more likely to have attendant disease (*16*). Finally, children 3–18 years of age have been reported to have similar risk for CDI as that seen in adults (*16*). Because the epidemiology of CDI is changing rapidly in children and adults, we examined age-specific trends in CDI-related hospitalizations in the US population <18 years of age.

## Materials and Methods

To characterize the epidemiology of CDI-related hospitalizations among US children, we performed 2 analyses using 2 databases. These databases are based on administrative coding, and consistency in results obtained from >1 data source potentially indicates a higher chance of accuracy. In addition, the format in which we analyzed 1 of the databases (Kids’ Inpatient Database [KID]) did not enable separating newborn discharges (defined as those hospitalizations during which the child was born) from those of other children <1 year of age. Because newborns represent a unique population prone to colonization but not overt disease, we chose a second database that enables separate analysis of newborns (National Hospital Discharge Survey [NHDS]).

The first analysis was a time-series analysis of all CDI-related hospitalizations among US children between 1997 and 2006 based on the data from KID within the Healthcare Cost and Utilization Project (HCUP) administered by the Agency for Healthcare Research and Quality (Rockville, MD, USA). This type of longitudinal analysis is useful for tracking disease patterns over long periods. KID was specifically designed to identify, track, and analyze national trends in healthcare use, access, charges, quality, and outcomes; in 2006, it included data from 3,739 hospitals from 38 states in the United States (*17*). Complex survey methods exist to develop national and regional estimates for conditions addressed in the database. The Agency for Healthcare and Research Quality assesses completeness and data quality, and documentation is provided with the dataset. Data quality checks are limited to logical issues (e.g., birth date precedes age at hospital admission, excessively low total charges or long length of stay, age <10 years or >55 years on a maternal record, and mixed neonatal and maternal records), i.e., no chart reviews are undertaken by the agency.

For the current study, all data were derived in aggregate from the publicly available HCUPNet website (*18*). Because the years for which data were available were 1997, 2000, 2003, and 2006, our observations were limited to these periods. We examined the annual incidence of CDI-related hospitalizations on the basis of the International Classification of Diseases, 9th revision, clinical modification (ICD-9-CM), code 008.45 (intestinal infection with *C*. *difficile*) as a proportion of all hospitalizations. We additionally determined the time trends for CDI as the principal discharge diagnosis in this population. Finally, to understand better the context of increasing CDI-related hospitalizations, we examined trends in hospitalizations related to other diarrheal diseases, specifically *Salmonella* (ICD-9-CM 00.30), rotavirus (ICD-9-CM 008.61), viral enteritis (ICD-9-CM 008.8), and other infectious enteritides (ICD-9-CM codes 009.0–009.3, 487.8).

The second analysis was a cross-sectional characterization of all CDI hospitalizations for patients <18 years of age in 2006 reported in the NHDS collected by the Centers for Disease Control and Prevention (Atlanta, GA, USA) and available as a public utility file from the National Center for Health Statistics (NCHS) (Hyattsville, MD, USA) (*19*). NHDS covers discharges from ≈500 noninstitutional, nonfederal, short-stay hospitals in the United States. The 3-stage survey design enables balanced geographic representation; data are abstracted either manually or electronically for an ≈1% representative sample of all US hospitalizations. To ensure quality and completeness of the data, NCHS at the Centers for Disease Control and Prevention conducted studies in the late 1970s. These surveys indicated that the NHDS data met the general standards for quality set by NCHS (*20*).

## Results

**Figure 1:**
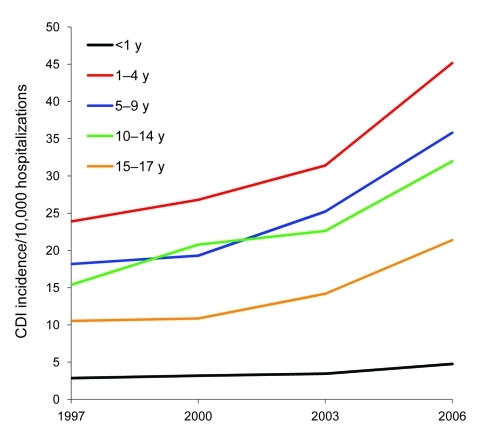
Age-specific incidence of patients with *Clostridium difficile* infection (CDI) per 10,000 hospitalizations, Health Care Utilization Project Kids’ and Inpatient Database, United States, 1997–2006.

**Figure 2:**
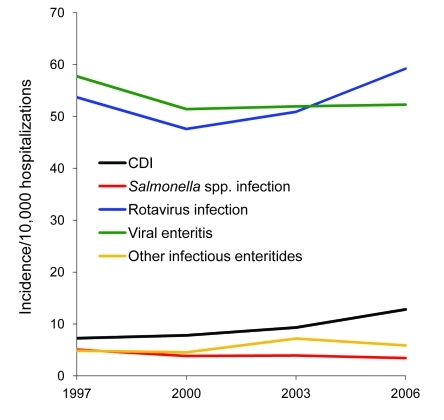
Incidence of infectious diarrhea hospitalizations per 10,000 all-cause hospitalizations, Health Care Utilization Project and Kids’ Inpatient Database, United States, 1997–2006. CDI, *Clostridium difficile* infection.

According to data from KID, the national number of pediatric CDI-related hospitalizations increased from 4,626 in 1997 to 8,417 in 2006 (Table 1). This change corresponded to an increased rate from 7.24/10,000 hospitalizations in 1997 to 12.80/10,000 hospitalizations in 2006 and represented a crude 9.0% per year increase. Although the group <1 year of age consistently accounted for the largest proportion of all pediatric CDI-related hospitalizations (Table 1), this group accounted for the lowest rate of CDI hospitalizations across the entire group of children; the highest incidence in all 4 years was detected in the group 1–4 years of age (Figure 1). The proportion of all CDI-related hospitalizations that had CDI listed as the principal discharge diagnosis remained essentially stable over the period examined (Table 1). Among other infectious gastroenteritis-related hospitalizations, only rotavirus had a similar upward trajectory during this period (Figure 2).

**Table 1:** *Clostridium difficile* infection–related hospitalizations, by year and age group, HCUP and KID, United States*

Characteristic	1997	2000	2003	2006
Age group, y				
<1	1,269	1,444	1,586	2,269
1–4	1,480	1,453	1,880	2,587
5–9	699	673	934	1,255
10–14	602	815	920	1,197
15–17	576	574	716	1,110
All	4,625	4,960	6,035	8,417
% CDIs as principal diagnosis	0.31	0.29	0.27	0.29

*HCUP, Health Care Utilization Project; KID, Kids’ Inpatient Database; CDIs, *C. difficile* infections. The sums of individual age groups may not add up to the number of total cases because numbers represent rounded values of weighted estimates.

**Figure 3:**
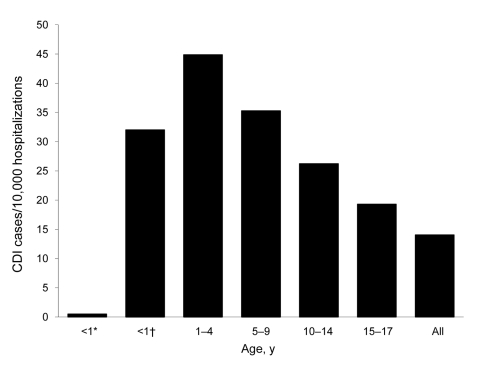
Age-specific incidence of *Clostridium difficile* infection (CDI) hospitalizations, National Hospital Discharge Survey, United States, 2006. *Newborn (i.e., during hospitalization for birth); †not newborn (i.e., during subsequent hospitalization).

Characteristics of CDI-related and nonrelated hospitalizations in the NHDS are shown in Table 2. The overall rate of pediatric CDI hospitalizations was 14.03/10,000 hospitalizations, and the age-specific rate did not differ substantially from that seen in the KID 2006 database (Figure 3). Although the newborn group, defined as infants whose hospitalizations originated at their birth, in NHDS represented 57.4% of all children hospitalized in 2006 and 84.2% of all children <1 year of age hospitalized, it had the lowest CDI rate of all pediatric age groups; the annual rate was 0.5/10,000 hospitalizations (Figure 3). Although the groups 1–4 and 5–9 years of age had the first (44.87/10,000) and second (35.27/10,000) highest rates of CDI hospitalizations, the third highest rate was seen in the non-newborn, <1 year of age group (32.01/10,000). The groups 10–14 and 15–17 years of age had the lowest rates of CDI hospitalizations in the pediatric cohort (Figure 3). There were no observed meaningful age-specific or sex-specific differences between the populations represented in the 2 data sources.

**Table 2:** Demographic characteristics for CDIs, National Hospital Discharge Survey, United States, 2006*

Characteristic	No. (%) CDIs	No. (%) non-CDIs
All pediatric hospitalizations	98	69,651
Age, y		
Newborn	2	40,022
<1 but not newborn	24	7,474
1–4	32	7,099
5–9	16	4,521
10–14	13	4,943
15–17	11	5,690
Sex		
M	48 (49.0)	35,941 (51.5)
F	50 (51.0)	33,808 (48.5)
Race		
Caucasian	59 (60.2)	39,457 (52.3)
African American	12 (12.2)	9,602 (13.8)
Other	7 (7.1)	5,589 (8.0)
Not stated	20 (20.4)	18,069 (26.0)

*CDIs, *Clostridium difficile* infections.

## Discussion

We found that CDI-related hospitalizations as a proportion of all hospitalizations among US children increased dramatically between 1997 and 2006, from 7.24 to 12.80/10,000 hospitalizations. Most of this increase occurred between 2000 and 2006, which possibly reflects spread of the new *C*. *difficile* strain into medical institutions. Consistent with finding from previous studies, children 1–4 years of age were as a group most likely to have a hospitalization that was CDI related, and newborns were the least likely. Such a low rate in newborns is consistent with long-standing recommendations against routine testing of children <1 year of age (*21*). This rate, rather than representing a truly low risk for CDI in this age group, may be the result of an inflated denominator, given that most births in the United States occur in a hospital setting. In contrast, non-newborn infants (i.e., those <1 year of age and not meeting the newborn definition) had the second highest rate of CDI-related hospitalizations. In addition to the overall increase in pediatric CDI-related hospitalizations, there was a coincident increase in hospitalizations either resulting from or complicated by rotavirus infection.

Several of these findings are consistent with other recent epidemiologic and microbiology-based investigations. For example, Klein et al. examined billing records for the testing of diarrheal stool specimens from children who came to the emergency department at a children’s hospital between 1998 and 2001 and identified *C*. *difficile* toxin in 6.7% (*8*). However, viral pathogens were isolated from 33% of the samples. A more recent study tracked changes between 2001 and 2006 in the epidemiology of *C*. *difficile* toxin testing performed on children at 1 academic medical center (*11*). The proportion of children <2 years of age who were positive increased from 46% to 64%, and there was a substantial increase in the incidence of community-onset infections and a concomitant decrease in hospital-onset infections. Interestingly, 43% of all patients had no recent history of exposure to antimicrobial drugs (*11*). Kim et al. estimated the rate of CDI in 22 US children’s hospitals and also found a steady increase from 4.4 cases/10,000 patient-days in 2001 to 6.5 cases/10,000 patient-days in 2006 (*9*).

The role of *C*. *difficile* in the pathogenesis of disease among non-newborn children <1 year of age remains perplexing. Because of historically low rates of pseudomembranous colitis (the characteristic pathologic lesion caused by toxins A and B) among infants and high rates of asymptomatic *C*. *difficile* carriage in neonates, it has been recommended that laboratory testing for CDI not be routinely performed for children <1 year of age (*21*). However, in the study by Kim et al., in which tests for *C*. *difficile* laboratory assays were combined with ICD-9-CM discharge diagnoses, 26% of all CDI cases were identified in infants and 5% in neonates (*9*). Although rates increased from 2001 through 2006 for children 1–5 years of age (from 0.7 to 1.3 cases/1,000 hospitalizations; p = 0.04) and those 5–17 years of age (from 1.2 to 1.8/1,000 hospitalizations; p = 0.03), these rates did not change for the group <1 year of age (from 3.1 to 3.0/1,000 hospitalizations).

Our data, which are more broadly representative of all pediatric admissions in the United States, have similar trends between 2000 and 2006 for children 1–4 years of age (from 2.68 to 4.52/1,000 hospitalizations) and those 5–17 years of age (from 1.62 to 2.86/1,000 hospitalizations). In contrast, the <1 year age group rates in our time series were an order of magnitude lower in 2000 and 2006. However, the rate for non-newborn children <1 year of age in our 2006 cross-sectional study (3.20/1,000 hospitalizations) was comparable with that observed by Kim et al. (*9*). The lower overall rate for children <1 year of age from our data likely reflects that healthy newborns have an exceedingly low risk for CDI and that although it is unlikely for these children to end up at a children’s hospital unless peripartum problems are encountered, neonates account for >80% of all hospitalized children <1 year of age in the HCUP and KID databases.

We could not determine whether the relatively high rate of CDI-related hospitalizations among non-newborn infants represents predominantly true disease or colonization. Although more specific than recovery of a toxin-producing strain from culture, even the detection of free toxins A, B, or both in the stool of a symptomatic infant does not ensure a pathogenic role for *C*. *difficile*, especially if another cause for diarrhea can be identified. Rates of hospitalizations for rotavirus infections have exhibited a similar increase as those with CDI between 1997 and 2006. Although 2 recent analyses of discharge data for adults suggest that non-CDI causes of diarrhea are not likely leading to a reporting bias as the explanation for the observed increase in CDI rates (*22*,*23*), the situation may be different for children in whom rotavirus is a serious pathogen and related hospitalizations are clearly increasing. Although Kim et al. did not report an increase in the frequency of testing for *C*. *difficile* in their study, our findings implicate this finding as a distinct possibility that needs to be investigated further (*9*).

Our study has several limitations. First, case identification was based on administrative coding, thus predisposing to misclassification. However, the degree of misclassification may not be substantial because multiple studies have shown the ICD-9-CM code 008.45 to be a relatively accurate way to identify CDI (*24*–*26*). Second, because we had no clinical data available, we could not distinguish stool colonization from CDI infection. Third, we were unable to distinguish community-acquired from healthcare-associated disease.

However, our study has several strengths. Because we explored 2 databases and discovered results that are highly consistent not only with each other but with those of previous recent investigations, we have augmented the accuracy of estimates of pediatric CDI incidence (*9*). In addition, our data are generalizable to most US-based institutions that care for the pediatric populations. This generalizability sets our results apart from those reported previously because they were limited to the highly specialized setting of children’s hospitals (*8*,*9*,*11*).

In summary, the incidence of CDI in the pediatric population appears to be increasing in US hospitals. A reporting bias for diarrheal diseases may play a role in this trend given the concomitant increase in rotavirus-related hospitalizations we identified. Future data may clarify this finding because widespread immunization with available rotavirus vaccines may soon lead to reduced incidence of related hospitalizations. The low incidence of CDI-related hospitalizations among newborns reflects current recommendations against routine testing and may support the concept that *C*. *difficile* does not cause disease among neonates. In contrast, the relatively high rate of CDI-related hospitalizations among non-newborn infants indicates an urgent need for studies to determine how often *C*. *difficile* causes true disease in this population.
